# Factors influencing the use of public dental services: An application of the Theory of Planned Behaviour

**DOI:** 10.1186/1472-6963-8-93

**Published:** 2008-04-30

**Authors:** Liana Luzzi, A John Spencer

**Affiliations:** 1 Australian Research Centre for Population Oral Health, School of Dentistry, The University of Adelaide, 5005, South Australia, Australia

## Abstract

**Background:**

There is limited evidence of the influence of psychosocial factors and health beliefs on public dental patient's patterns of service use in Australia. The research aims were to examine associations between dental attitudes and beliefs of public dental service users and dental visiting intention and behaviour using the Theory of Planned Behaviour.

**Methods:**

517 randomly selected adult public dental patients completed a questionnaire assessing dental attitudes and beliefs which was matched with electronic records for past and future dental service use. A questionnaire measured intentions, attitudes, subjective norms and perceptions of behavioural control and self-efficacy in relation to visiting public dentists. A measure of dental attendance at public dental clinics was obtained retrospectively (over 3 1/2 years) and prospectively (over a one year period following the return of the questionnaire) by accessing electronic patient clinical records.

**Results:**

Participants had positive attitudes, subjective norms and self-efficacy beliefs towards dental visiting but perceived a lack of control over visiting the dentist. Attitudes, subjective norms, self-efficacy and perceived control were significant predictors of intention (P < 0.05). Intentions, self-efficacy and past dental attendance were significant predictors of actual dental attendance (P < 0.05).

**Conclusion:**

Public dental patients held favourable attitudes and beliefs but perceived a lack of control towards dental visiting. Reducing structural barriers may therefore improve access to public dental services.

## Background

Regular, preventive dental attendance is a contributor to the oral health status of people of all ages. Studies have shown that preventive dental care leads to better oral health outcomes and gains in quality of life [1]. However, while the positive effects of regular, preventive dental visiting are well established in all age groups, there is evidence to demonstrate that many people do not attend the dentist regularly enough, particularly financially disadvantaged adults in the Australian population who rely on public dental services. While the majority of dental care in Australia is provided in the private sector, patients who attend for public care remain a public health focus due to their socioeconomic disadvantage [2]. Virtually all aspects of oral disease measured in the Australian 2004–06 National Survey of Adult Oral Health were more frequent and more severe among people who were eligible for public dental care [3]. According to this study, while there have been improvements in oral health, particularly among the 'fluoride generation' of Australians born since 1970, inequalities in oral health exist among those who have regular visits to a dentist and those who visit a dentist irregularly or only when a oral health problem arises, with the latter group worse off on almost all measures of oral health. Studies have shown that regular dental attendance is more prevalent among groups with higher socioeconomic status [4]. Furthermore, regular dental visits have been found to have a significant and positive effect on dental health, with not only the severity and prevalence of dental health problems significantly less among regular attenders, but also the experience of social and psychological consequences of dental health problems [5,6].

In order to help understand health behaviours, social cognition models have been developed and adopted in behavioural science research. These models endeavour to identify and explain how expectations, judgments, beliefs and intentions lead to the performance of various behaviours [7,8]. Research has shown that beliefs, attitudes, and knowledge; physical and social environments; and skill or control over performance of behaviours determine and limit health behaviours [9-11]. A widely used social cognition model is the Theory of Planned Behaviour (TPB) [12,13]. It has been used successfully to provide a better understanding and explanation of a diverse range of health-related and social behaviours [14-16], including addictive behaviours (e.g., smoking, alcohol consumption and drug use), clinical and screening behaviours (e.g., health checks and cancer screening), eating behaviours (e.g., healthy diets), exercising behaviours, HIV/AIDS-related behaviours (e.g., condom use) and oral hygiene behaviours (e.g., brushing and flossing teeth). Godin and Kok [15] reviewed 58 health behaviour studies and found that, on average, the model explained 41% and 34% of the variance in intention and behaviour respectively.

**Figure 1:**
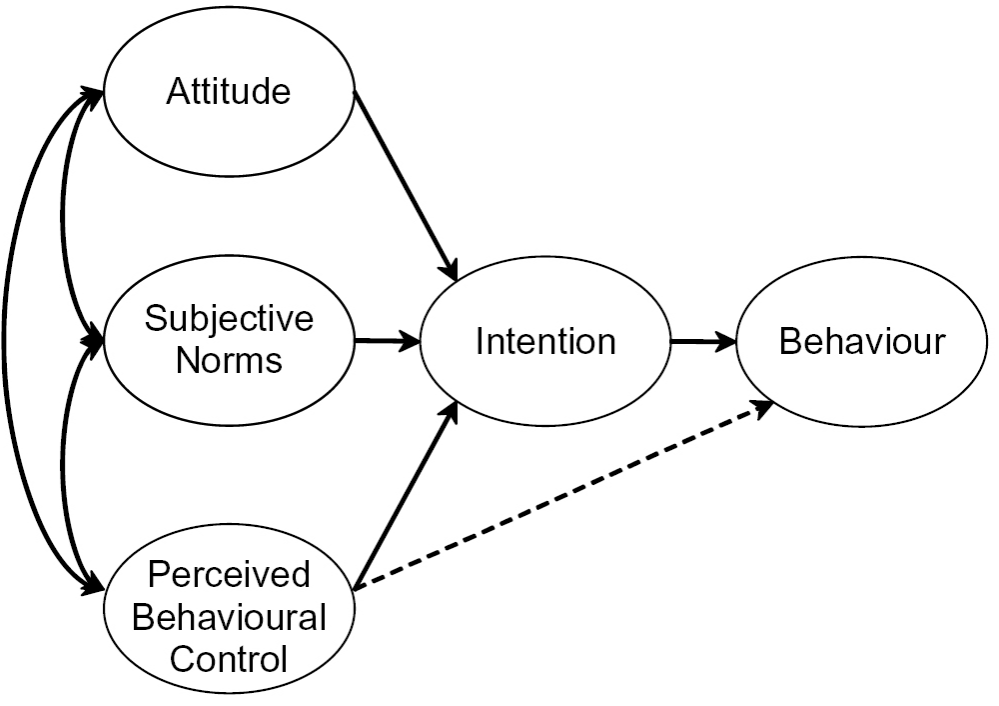
Theory of Planned Behaviour (Ajzen, 1991).

The TPB model postulates that behaviour is predicted by intention to perform the behaviour and also by perceived behavioural control when behaviour is not under complete volitional control. Intention to perform the behaviour is determined by the relative importance of three factors: (1) attitude toward the behaviour (i.e., a favourable or unfavourable evaluation of the behaviour); (2) subjective norm (i.e., perceived social pressure to perform or not to perform the behaviour); and (3) perceived behavioural control (i.e., perception of the extent to which the behaviour is within his or her control measured in terms of self-efficacy and controllability in relation to the behaviour). Figure 1 shows the basic conceptual model whereby the theory hypothesises that attitudes, subjective norms and perceived behavioural control have a direct effect on intentions and an indirect effect on behaviour through intentions. In addition, perceived behavioural control has a direct effect on behaviour. In general, the more favourable the attitude and the subjective norm, and the greater the perceived control the stronger one's intention is to perform the behaviour. The relationship between intentions and behaviour indicates that people are likely to carry out behaviours they intend to perform. The relationship between perceived behavioural control and behaviour not only suggests that people are more likely to perform favourable behaviours they have control over, but also that people are prevented from carrying out behaviours over which they have no control [14,17]. As described by Ajzen [13,18], these factors (i.e., attitude, subjective norm and perceived behavioural control) can be traced to corresponding sets of behaviour-related beliefs that reflect the underlying cognitive structure – (1) *behavioural beliefs*, which are assumed to influence attitudes toward the behaviour (i.e., beliefs about the likely outcomes of the behaviour and the evaluations of these outcomes); (2) *normative beliefs*, which constitute the underlying determinants of subjective norms (i.e., beliefs about the normative expectations of others and motivation to comply with these expectations); and (3) *control beliefs*, which provide the basis for perceptions of behavioural control (i.e., beliefs about the presence of factors that may facilitate or impede performance of the behaviour and the perceived power of these factors). By measuring these beliefs there is an opportunity to gain a better understanding of how attitudes, subjective norms and perceived behavioural control are formed. This, in turn, would enable a better understanding of why individuals do or do not use dental services. For example, attitudes stem from evaluations of potential consequences that can result from performing the behaviour. If the underlying behavioural beliefs related to these consequences are favourable, a favourable attitude results and an individual is likely to visit the dentist. Thus, belief structures underlying attitude, subjective norm and perceived behavioural control can be used to design educational programs among adults attending public dental services. These beliefs can provide information about what to include in persuasive measures to increase intention with respect to preventive dental attendance and can be extremely useful in the design and implementation of effective programs of behavioural intervention [18].

In addition to using the major constructs of the TPB, past dental attendance behaviour within the public dental system was also examined as a potential predictor of intentions and future behaviour. Some studies have found that the inclusion of past experience contributed significantly to the prediction of behavioural intentions [19,20]. A similar conclusion was reached by Sheeran and Taylor [21] in their meta-analysis. Recently, Ajzen [17] revised TPB, by theoretically proposing linkages between past behaviour and future behavioural intent. In addition, numerous studies have shown that there is a direct relationship between measures of past and future behaviour, even after controlling for intention and perceived behavioural control [22,23]. Past behaviour is thought to have a moderating effect on one's perceptions of the control they have over the behaviour, since having performed the behaviour in the past may alter their perceptions of being able to perform it in the future [12]. Once one believes that they are able to perform a particular behaviour, there is an increased possibility of performing the behaviour in the future. This notion supports Bandura's [24] self-efficacy theory.

A study by Woolgrove, Cumberbatch and Gelbier [25] used the earlier model, Theory of Reasoned Action (a model used to explain behaviours under complete volitional control) to evaluate the factors that influence regular dental attendance among individuals in their mid-teens. And while a number of studies have adopted the TPB in the prediction of various clinical and screening behaviours (e.g., Norman and Conner [26]), there have been few, if any, empirical studies using the TPB in the area of dental visiting among adults. The objectives of this research were therefore: (1) to predict dental visiting intentions by examining the influence of attitudes, subjective norms and perceived behavioral control on intention to visit the dentist; (2) to predict dental visiting behaviour by examining the influence of intention, perceived behavioural control and past dental attendance behaviour on actual dental attendance; and (3) to explore the cognitive and affective foundation (i.e., belief structures) that is assumed to determine dental visiting intention and hence dental attendance behaviour.

## Methods

### Participants

This research used an existing and established random sample of 893 public dental patients recruited across public dental clinics in SA using a prospective, cohort design [27]. Participants were informed of the study at the time they contacted the clinic for dental care. The selection criteria used were that participants had to be aged 18 years or more, be dentate with 6 or more natural teeth, and be a holder of a government concession card entitling them to public dental care. Those seeking general dental care had to be new to the waiting list, and should not have visited a dentist (private or public) for routine dental in the last 12 months care (patients who received emergency dental care in the last 12 months were included). Upon being recruited, participants received either a course of emergency dental care or general dental care.

### Questionnaire

The questionnaire contained items designed to assess the main constructs in the TPB model and was based on the methodology described by Ajzen [12,13]. Intention, attitude, subjective norm and perceived behavioural control in relation to visiting a public dentist/using the public dental service were each assessed by means of several direct questions. The perceived behavioural control construct was further broken down into the two components self-efficacy (reflecting internal aspects of control) and perceived control (reflecting external aspects of control) as there is substantial empirical evidence supporting the multi-dimensional nature of this construct [28-32]. Beliefs postulated to provide the cognitive foundations for attitude, subjective norm and perceived behavioural control were also assessed. In order to know what sorts of beliefs to include in the questionnaire, structured interviews were conducted with a sample of 20 adult public dental patients attending public dental service clinics in SA. Patients were questioned about the advantages and disadvantages of using the public dental service *(to identify relevant outcomes)*, the people important to them who would approve or disapprove of them using the public dental service *(to identify relevant referents) *and, the factors or circumstances that would make it easier or more difficult for them to use the public dental service *(to identify relevant control factors)*. Some 18 relevant outcomes, five relevant referents and eight relevant control factors were identified (Table 1).

**Table 1:** Relevant outcomes, referents and control factors related to dental visiting

**OUTCOMES**	
Prevent tooth decay	Receive unnecessary extractions
Keep teeth healthy	Prevent future problems with teeth, mouth or dentures
Have teeth cleaned	Receive dental advice from dental professional
Keep teeth looking good	Get dental problems fixed if there were any problems to be fixed
Prevent pain in teeth, mouth or dentures	Have to wait a long time in waiting room for appointment
Have good oral health	Experience painful dental treatment
Receive preventive treatments	Seen promptly
Prevent tooth loss	Afraid about dental visit
Receive fillings to fix dental decay	Anxious about dental visit
	
**REFERENTS**	
Family	Mother
Partner	Friend/s
Parent/s	
	
**CONTROL FACTORS**	
Long waiting lists^†^	Being afraid about the dental visit^‡^
Costly dental treatment^†^	Being anxious about the dental visit^‡^
Having to pay a gap, i.e., co-payments^†^	Convenient location of dental clinic^†^
Bad dental experience^‡^	Having to pay for dental treatment, regardless of amount^†^
Not having choice of dentist^†^	

^†^external control factors.

^‡^internal control factors

### Direct measures

*Behavioural intention *to visit the dentist was assessed using the following two items, each rated on 7-point *disagree-agree *scales: 'I want to visit the dentist' and 'I plan to visit the dentist'. *Attitude *was measured using two evaluative semantic differential scales (*harmful-beneficial *and *worthless-worthwhile*) in response to the item: 'My visiting the dentist would be...'. *Subjective norm *was measured using the following three items, each rated on 7-point *disagree-agree *scales: 'People who are important to me think that I should visit the dentist', 'People who are important to me would approve of me visiting the dentist', and 'People who are important to me want me to visit the dentist'. *Perceived behavioural control *was assessed from responses to five items, two of which assessed self-efficacy while the other three assessed perceived control. The two items used to assess self-efficacy were: 'For me to visit the dentist from now on would be...' using a 7-point *difficult-easy *scale and 'What is the likelihood of you visiting the dentist from now on?' using a 7-point *unlikely-likely *scale. The three items used to measure perceived control were: 'Whether or not I visit the dentist is entirely up to me', 'It is mostly up to me whether I visit the dentist', and 'I have complete control over whether or not I visit the dentist', each using a 7-point *disagree-agree *scale.

Direct measures of behavioural intention, attitude, subjective norm, self-efficacy and perceived control were obtained by averaging the responses to items measuring each respective factor. For each measure the possible range of mean scores was 1 to 7. All the variables were scored consistently so that higher mean scores reflected more positive attitudes towards dental visiting, more positive subjective norms to visit the dentist, and higher perceived behavioural control to visit the dentist.

For each construct, there were very few missing values, with 95.2%, 96.9% 98.5%, 97.5% and 97.7% of respondents providing responses to all items measuring intention, attitude, subjective norm, self-efficacy and perceived control respectively. In the few cases where missing values were encountered, those who provided no response to any of the items for the various constructs were excluded from analyses.

### Belief-based measures

#### Behavioural beliefs

Respondents were asked to indicate their agreement with the 18 identified outcomes to visiting the dentist (e.g. 'I think that by visiting the dentist I will prevent decay in my teeth' *(unlikely, -3 to likely, +3)*) and their evaluation of the outcomes (e.g. 'Preventing tooth decay is...' *(bad, -3 to good, +3)*). Responses to each pair of items were multiplied together and a behavioural belief scale (i.e., a belief-based measure of attitude) was subsequently constructed by summing these products across all beliefs [33]. The range of possible scores was -162 to +162.

#### Normative beliefs

Respondents were asked to indicate the extent to which the 5 identified referents would approve or disapprove of them visiting the dentist (e.g. 'To what extent would your family disapprove or approve of you visiting the dentist?' *(disapprove, -3 to approve, +3)*) and the extent to which they were motivated to comply with these referents (e.g. 'Generally speaking, I would like to do what my family thinks I should do' *(not at all, +1 to very much, +7)*). Responses to each pair of items were multiplied together and a normative belief scale (i.e., a belief-based measure of subjective norm) was subsequently constructed by summing these products across all referents [33]. The range of possible scores was -105 to +105.

#### Control beliefs

Respondents indicated the extent to which the 8 identified control factors would facilitate or hinder dental visiting (e.g. 'How difficult or easy for you would long waiting lists make visiting the dentist?' *(difficult, -3 to easy, +3)*) and their likelihood of occurrence (e.g. 'If I were to visit the dentist I expect that there would be long waiting lists' *(disagree, -3 to agree, +3)*). Responses to each pair of items were multiplied together and a belief-based measure of perceived behavioural control was constructed by summing these products across all control beliefs [33]. The range of possible scores was -72 to +72. A belief-based measure of self-efficacy and perceived control was also obtained as three of the eight control beliefs represented self-efficacy concerns and five perceived control concerns. Self-efficacy and perceived control belief scales were constructed in a similar fashion to the other scales and the range of possible scores was -27 to +27 and -45 to +45 respectively.

For each of the respective belief-based measures, a positive score indicated a favourable attitude toward dental visiting, positive social pressure to visit the dentist and a feeling of being in control over visiting the dentist, while a negative score indicted the opposite.

### Data collection methodology

The data collection methodology was based on the 'Total Design Method' [34]. All patients in the sample were sent a primary approach letter, followed by the questionnaire a week later. A reminder card and two-follow-up mailings were sent to non-respondents. Approval to conduct this research was granted by The University of Adelaide Human Research Ethics Committee and the SA Dental Service Ethics Committee.

### Target behaviour

The behaviour of interest was actual dental attendance behaviour post-questionnaire, defined as either 'Yes, visited post-questionnaire' or 'No, did not visit post-questionnaire'. Information on actual dental attendance was obtained by accessing electronic patient clinical records just over a year after the questionnaire mail-out.

In accordance with the Principle of Compatibility [12,35,36] (i.e., all the measures in the questionnaire – attitude, subjective norm, perceived behavioural control, and intention – should refer to the same level of generality or specificity to maximise their relationship with behaviour), the behaviour of interest in this research was defined carefully in terms of its Target, Action, Context, and Time (TACT) elements. Thus, the target behaviour was defined in terms of its four elements: 1) action – visiting the dentist; 2) target towards which the action is directed – the public dentist; 3) the context (or setting) in which it occurs – at a public dental clinic; and 4) time at which it is performed- during the eligibility period. The time element used for this research was very general/broad. This research was primarily interested in persons using/attending the public dental service as an eligible adult. The timeframe here therefore encompassed the time period in which a person was eligible to use public dental services (i.e., a dental visit could potentially be made any time up until the expiry date of their government concession card). Intentions, attitudes, subjective norms and perceived behavioural control were subsequently measured within this time period.

### Past behaviour

Past dental attendance of the sample was obtained retrospectively by accessing electronic patient clinical records, and was defined based on the type(s) of course of care (CoC) (i.e., emergency or general CoC) patients received over a period of up to 3 1/2 years. Patients were classified as either an emergency attender, general attender or some combination of the two. Those persons for which some or all of the CoC they received were not able to be determined were excluded from analyses.

### Analysis

The factor structure of the TPB was tested using confirmatory factor analysis. Internal reliability of the direct measures was assessed using Cronbach's alpha, α. Descriptive analysis was conducted to describe the distributions of the cognitive measures. Correlations between the belief-based measures and their corresponding direct measures were examined to determine if the appropriate beliefs were identified and properly measured. Multiple linear regression analysis was used to model the relationship between intention and the direct measures. Binary logistic regression was used to model the relationship between actual dental attendance and intention, self-efficacy, perceived control and past behaviour. Adjusted odds ratios were determined from this model of actual dental attendance post-questionnaire, with the dependent variable coded as 1 if persons visited post-questionnaire with the reference category of 'did not visit post-questionnaire' coded as 0.

## Results

### Sample characteristics

Overall 517 persons responded to the survey (adjusted response rate= 67.4%). The mean age of participants was 54.9 years (± 16.3 years), 60.0% of participants were female, 57.6% were born in Australia, 90.2% mainly spoke English at home, 99.2% were non-Indigenous and 10.4% had private dental insurance.

### Factor analysis and reliability analysis

In a four-factor solution, items measuring each cognitive construct loaded highly on the same factor (loadings of 0.79 or higher) and had negligible loadings on the remaining three factors (loadings of 0.01 or lower). The direct measures of intention, attitude, subjective norm, self-efficacy and perceived control exhibited a reasonable level of internal consistency, with α coefficients of 0.73, 0.74, 0.89, 0.64 and 0.91 respectively. Internal reliability measures are not appropriate for the belief-based measures as they are formative rather than reflective indicators of the measured construct [37,38].

### Descriptive statistics and correlations

Means for each of the direct cognitive measures were quite high, indicating strong intention and favourable attitude, subjective norm and perceived behavioural control toward visiting the dentist (Table 2). Intention had a statistically significant positive correlation with attitude, subjective norm and self-efficacy, but was not correlated with perceived control. The other components of the model were also significantly correlated with each other. Consider for example the correlation between attitude and intention which was 0.32. This was of moderate strength and statistically significant. Intuitively, this means that the more positive a person's attitudes, the more likely they are to intend to visit the dentist. However, this correlation leaves considerable room for other influences on intentions. Attitude certainly is not the only important predictor of intention. Positive attitudes towards visiting the dentist do not necessarily translate into intention to do so. As suggested by the TPB, and by the results obtained from the correlation analyses (Table 2), subjective norm and perceived behavioural control are also important predictors of intentions to visit the dentist.

**Table 2:** Cognitive measures (direct and belief-based): means, standard deviations, and correlations (n = 517)

				**Correlation coefficients**^†^					
				**Direct measures**					
				
	**n**	**Mean**	**SD**	**INT**	**ATT**	**SN**	**PBC**	**SE**	**PC**
**Direct measures**^‡^									
Behavioural Intentions (INT)	501	5.526	1.568	-					
Attitudes (ATT)	509	6.290	0.713	0.321**	-				
Subjective norms (SN)	511	5.665	1.348	0.284**	0.381**	-			
Perceived behavioural control (PBC)	510	5.699	1.248	0.209**	0.228**	0.210**	-		
-Self-efficacy (SE)^#^	510	5.235	1.341	0.287**	0.285**	0.241**	0.779**	-	
-Perceived control (PC)^#^	508	6.011	1.615	0.068	0.107*	0.094*	0.787**	0.312**	-
**Belief-based measures**									
Behavioural beliefs^¥^	514	59.224	42.940	0.196**	0.353***				
Normative beliefs^§^	498	35.203	30.791	0.104*		0.415***			
Control beliefs^^^	513	-8.653	15.330	0.017			0.262***		
-Self-efficacy beliefs^¶^	380	-2.763	6.561	0.054				0.344***	
-Perceived control beliefs^∞^	511	-6.632	13.200	-0.024					0.115***

***P < 0.0001, **P < 0.01, *P < 0.05

^† ^Spearman's rho

^‡ ^1 ≤ Mean score ≤ 7

^# ^SE and PC are 2 distinct components derived from the PBC factor

^¥ ^-162 ≤ Behavioural belief score ≤+162

^§ ^-105 ≤ Normative belief score ≤ +105

^^ ^-72 ≤ Control belief score ≤ +72

^¶ ^-27 ≤ Self-efficacy beliefs score ≤ +27

^∞ ^-45 ≤ Perceived control beliefs score ≤ +45

### Distribution of belief-based measures

The mean behavioural belief score reflected moderately positive attitudes towards dental visiting and the average normative belief score reflected fairly weak positive social pressure to visit the dentist. The mean control belief score indicated a degree of negativity about control, suggesting that visiting the dentist was somewhat difficult for study participants (both in terms of self-efficacy and perceived control concerns) (Table 2).

### Associations between belief-based and direct measures

Each set of beliefs was significantly correlated with their direct measure (Table 2). The behavioural beliefs score had a correlation of 0.35 (P < 0.0001) with the direct measure of attitude, suggesting that the set of behavioural beliefs captured overall attitudes moderately well. The normative beliefs score had a correlation of 0.42 (P < 0.0001) with the direct measure of subjective norm, indicating a reasonably moderate strength of relationship between the two measures. The control beliefs, however, did not correlate as strongly with the direct measure of perceived behavioural control. The correlation between the control beliefs score and the direct measure of perceived behavioural control was 0.26 (P < 0.0001). It appears the control factors identified in the structured interviews did not capture well all the important considerations related to perceived behavioural control. However, when the belief-based measure of self-efficacy was correlated against the direct measure of self-efficacy, the correlation coefficient was 0.34 (P < 0.0001), indicating that the set of self-efficacy beliefs were stronger in capturing the overall feeling of self-efficacy. However, the correlation between the belief-based and direct measure of perceived control was only 0.12 (P < 0.0001), indicating that the external control factors were only weakly associated with a person's perception of the control they have over visiting the dentist.

### Target behaviour

Questionnaire respondents were followed for an average of 1.17 years (± 0.07 years) in order to track their actual dental visiting behaviour post-questionnaire and to ensure that the measure of behaviour obtained could be directly related to their reported dental visiting intention. Overall, 35.4% (183 out of 517 questionnaire respondents) made a dental visit after completing the questionnaire.

### Past behaviour

Among those who returned the questionnaire (*n *= 493), 32% did not visit within the 3 1/2 year period, 36.9% had a past pattern of emergency dental visiting, 9.1% had a past pattern of general dental visiting and 21.9% had a past pattern of both emergency and general dental visiting.

### Predicting behavioural intentions

In order to identify the most important predictors of dental visiting intention, sex, age and the TPB variables were entered into a hierarchical regression analysis. Age and sex together were able to explain only 0.5% of the variance in intentions to visit the dentist (F = 2.312, d.f. = 489, P = 0.100). The addition of the TPB variables led to a significant increase in the amount of variance explained (R^2 ^change = 0.127, F change = 16.87, P < 0.0001). Past behaviour was only able to explain 0.5% of the variance in behavioural intentions (R^2 ^change = 0.001, F change = 0.111, P = 0.954), and did not emerge as a significant, independent predictor of intention, contributing negligibly to any additional variance. Together the variables under consideration were able to explain 12.0% (adjusted-R^2^) of the variance in intention (F = 8.035, d.f. = 466, P < 0.0001). All four TPB variables emerged as significant independent predictors in the final regression equation. Public dental patients who intended to visit the dentist were more likely to have positive attitudes toward visiting the dentist, perceive positive social influences and have confidence in their ability to visit the dentist. However, public dental patients intending to visit the dentist were less likely to believe that they had control over their dental visiting. Examination of the squared semi-partial correlations revealed that attitude explained 3.1% of the unique variance in intention, subjective norm 1.4%, self-efficacy 3.3% and perceived control 0.8%. Self-efficacy concerns appeared to be the most important predictor of intention, followed by attitude (Table 3).

**Table 3:** Multiple linear regression analysis to predict dental visiting intentions among adult public dental patients (n = 490)

**Model**^†^	**β**^‡^	**SE**^‡^	**P-value**	**Semi-partial correlation squared**
(Constant)	1.402	0.702	0.046	
Age	-0.002	0.005	0.742	0.020
Sex	-0.005	0.142	0.974	0.000
Direct attitude^#^	0.425	0.103	0.000	3.125
Direct subjective norm^#^	0.152	0.055	0.006	1.426
Direct self-efficacy^#^	0.250	0.060	0.000	3.285
Direct perceived control^#^	-0.103	0.049	0.035	0.843
Past dental attendance behaviour*				
-Emergency attender	-0.084	0.167	0.617	0.047
-General attender	0.017	0.260	0.948	0.001
-Emergency and General attender	-0.022	0.196	0.912	0.002

F(9,457) = 8.053 (P < 0.0001) ^†^Dependent variable = behavioural intentions mean score *Reference: Did not visit within the 3 1/2 year period ^‡^unstandardised ^#^mean score				

**Step**	**R**	**R**^2^	**Adjusted- R**^2^	

1^¥^	0.093	0.009	0.007	
2^§^	0.097	0.009	0.005	
3^^^	0.298	0.089	0.083	
4^¶^	0.316	0.100	0.092	
5^∞^	0.350	0.122	0.113	
6^≈^	0.361	0.130	0.119	
7^+^	0.370	0.137	0.120	

^¥^Age

^§^Age, Sex

^a^^Age, Sex, Direct attitude

^¶^Age, Sex, Direct attitude, Direct subjective norm

^∞^Age, Sex, Direct attitude, Direct subjective norm, Direct self-efficacy

^≈^Age, Sex, Direct attitude, Direct subjective norm, Direct self-efficacy, Direct perceived control

^+^Age, Sex, Direct attitude, Direct subjective norm, Direct self-efficacy, Direct perceived control, Past behaviour

Correlational analyses (Table 4) further revealed which beliefs played an important role in influencing one's intention to visit the dentist (Spearman's rho, P < 0.05). The beliefs underlying attitudes that were most strongly connected to intentions were with 'keep teeth healthy', 'prevent future problems' and 'have good oral health'. This indicated that people were first and foremost looking at the experience of visiting the dentist in terms of longer-term oral health outcomes. In addition, outcomes such as 'prevent tooth decay', 'keep teeth looking good', 'prevent pain in teeth, mouth or dentures', 'receive preventive treatments', 'receive fillings to fix dental decay' and 'prevent loss of teeth' were also among the beliefs to have a higher correlation with dental visiting intentions. Not being dentally anxious, having teeth cleaned, receiving dental advice from a dental professional and being seen promptly were also important correlates of intentions. The more that people thought that visiting the dentist offered these things, the more likely they were to say that they intended to visit the dentist. Thus, strengthening these perceptions should also strengthen people's intention to visit the dentist. Commonly cited 'downsides' of visiting the dentist appeared only weakly to undermine intentions. These included receiving unnecessary extractions, having to wait a long time in the waiting room for the scheduled appointment, experiencing painful dental treatment and being dentally afraid. These 'downsides' to visiting the dentist tended to put respondents off only slightly, and this was more than counteracted by the extent to which the positive beliefs encouraged them to consider visiting the dentist. Respondent's perceptions of the opinions and support of their family, parent/s, mother, and friend/s were correlated with their intention to make a dental visit. Therefore, the greater the perceived positive support from these people to visit the dentist, the more likely respondents were to intend to visit the dentist. The belief about there being long waiting lists had a significant negative correlation with intentions, indicating that there was a tendency for respondents who saw this factor as a barrier to have a weaker tendency not to intend to visit the dentist.

**Table 4:** Beliefs and their correlations with behavioural intention

**Beliefs**	**Correlation**^#^	
***Behavioural beliefs***	***b***_*i*_***e***_*i*_***with BI***	
Prevent tooth decay	0.174	**
Keep teeth healthy	0.203	**
Prevent future problems with teeth, mouth or dentures	0.179	**
Keep teeth looking good	0.133	**
Prevent pain in teeth, mouth or dentures	0.144	**
Have good oral health	0.197	**
Receive preventive treatments	0.126	**
Have teeth cleaned	0.133	**
Receive fillings to fix dental decay	0.160	**
Receive unnecessary extractions^†^	0.035	
Prevent loss of teeth	0.136	**
Receive dental advice from a dental professional	0.100	*
Get dental problems fixed if there were any problems to be fixed	0.061	
Have to wait a long time in the waiting room for the appointment^†^	0.017	
Experience painful dental treatment^†^	0.036	
Seen promptly	0.093	*
Afraid about the dental visit^†^	0.080	
Anxious about the dental visit^†^	0.139	**
***Normative beliefs***	***nb***_*j*_***mc***_*j*_***with BI***	
Family	0.118	*
Partner	0.102	
Parent/s	0.163	**
Mother	0.140	*
Friend/s	0.119	*
***Control beliefs***	***c***_*k*_***p***_*k*_***with BI***	
Long waiting lists	-0.098	*
Costly dental treatment	0.026	
Having to pay a gap, i.e., co-payments	0.001	
Bad dental experience^†^	-0.006	
Not having choice of dentist	-0.073	
Being afraid about the dental visit^†^	0.027	
Being anxious about the dental visit^†^	0.109	
Inconvenient location of dental clinic	0.031	

BI = Behavioural intention

# Spearman's rho correlation coefficient

† item corrected for direction of response

b_i_e_i _= Behavioural belief strength × outcome evaluation, i = 1 to 18

nb_j_mc_j _= Normative belief strength × motivation to comply, j = 1 to 5

c_k_p_k _= Control belief strength × control power, k = 1 to 8

* Correlation is significant at the 0.05 level (2-tailed) (P < 0.05)

** Correlation is significant at the 0.01 level (2-tailed) (P < 0.01)

### Predicting behaviour

In order to predict actual dental attendance behaviour, intention, self-efficacy, perceived control, past behaviour, age and sex were entered as predictor variables into a binary logistic regression model. Intention, self-efficacy, past behaviour and age emerged as significant predictors of actually having made a dental visit post-questionnaire, but perceived control and sex were not significant predictors (Table 5). The estimated odds of having made a dental visit post-questionnaire increased by 18.4% and 22.9% when mean intention and self-efficacy scores respectively increased by one. In addition, those who had a past pattern of emergency dental visiting had 2.7 times the odds of making a dental visit post-questionnaire compared to those who did not visit within the 3 1/2 year period. Those who had a past pattern of both emergency and general dental visiting had 2.2 times the odds of making a dental visit post-questionnaire compared to those who did not did not visit within the 3 1/2 year period. Also, older persons had slightly greater odds of making a dental visit post-questionnaire. Overall, the amount of variance in actual dental attendance explained by the predictor variables in the model was 15.5%. The addition of a measure of past behaviour increased the amount of variance explained by 7.0% (Table 5).

**Table 5:** Binary logistic regression analysis to predict actual dental attendance among adult public dental patients (n = 470)

**Model**^†^	**Beta**	**OR**	**95% CI for OR**	**P-value**
Age	0.016	1.016	(1.002,1.030)	**0.022**
Sex*	0.121	1.128	(0.744,1.711)	0.570
Behavioural intention^‡^	0.169	1.184	(1.027,1.364)	**0.020**
Direct self-efficacy^‡^	0.206	1.229	(1.021,1.479)	**0.029**
Direct perceived control^‡^	-0.020	0.980	(0.847,1.133)	0.783
Past dental attendance behaviour^#^				**0.000**
-Emergency attender	1.011	2.749	(1.659,4.555)	0.000
-General attender	-0.486	0.615	(0.254,1.489)	0.281
-Emergency and General attender	0.801	2.228	(1.256,3.952)	0.006
Constant	-4.050	0.017		0.000

**Step**	**NagelkerkeR**^2^	**ΔR**^2^		

1^¥^	0.043	--		
2^§^	0.067	0.024		
3^^^	0.085	0.018		
4^~^	0.155	0.070		

^†^Dependent variable = Actual dental attendance post-questionnaire (Yes, visited (coded as 1) vs. No, did not visit (coded as 0))

^‡^mean score

^#^Reference: Did not visit within the 31/2 year period

*Reference: Male

^¥^Age, sex

^§ ^Age, sex, Behavioural intention

^^ ^Age, sex, Behavioural intention, Direct self-efficacy, Direct perceived control

^~^Age, sex, Behavioural intention, Direct self-efficacy, Direct perceived control, Past dental attendance behaviour

## Discussion

### Representativeness

Despite achieving a reasonably high response rate to the questionnaire, non-response analyses were conducted to determine whether those who responded were representative of the total sample. Age, sex and baseline sample type (i.e., participants were originally classified based on the nature of the course of care they received at a public dental clinic at the time they were recruited, i.e., as either an emergency or general dental care patient) were examined between responders and non-responders. No significant differences were found between the proportions of responders and non-responders in terms of sex and baseline sample type (χ^2 ^test, P > 0.05). However, age differed significantly between those who responded and those who did not respond to the questionnaire, with responders being significantly older on average (55.9 years cf. 44.7 years; ANOVA, P < 0.0001). Thus the sample of responders was not totally representative of the age distribution of the total sample. However, in the regression model predicting behavioural intention where age was controlled for, age did not emerge as a significant independent predictor of intentions so in terms of the prediction of intention, it can be assumed that no significant biases were introduced in the sample. However, in the model predicting actual dental attendance, age was a significant independent predictor behaviour, so with respect to the assessment of behaviour, some bias may have been introduced the sample. That said however, in the National Survey of Adult Oral Health conducted in 2004–06 [3], the average age of dentate adults eligible for public funded dental care was found to be 54.0 years in South Australia and 52.7 years nationally, so the age distribution of the sample used in this research does not differ very much from that in the National survey.

Patients' records were accessed electronically which enabled the collection of data relating to service use during the 3-year follow-up period and post-TPB questionnaire for the majority of participants in the sample. Databases across community dental clinics in South Australia were cross-matched with patient details to ensure that if a patient visited public dental clinics at different locations across the follow-up period, their data relating to visits and service provision would be identified and extracted. Therefore, most of, if not all, data related to public dental visitations was captured.

Whilst it is possible that some patients may have attended a private dental practice for dental care, data examining this group of patient's use of private dental services over the follow-up period was not collected. One of the main purposes behind following these patients was to determine their dental attendance behaviour based on a pattern of attendance *in the public sector *during the follow-up period. The questionnaire itself was designed to explain and predict dental visiting behaviour within the public sector, and so measures of intentions, attitudes, subjective norms and perceived behavioural control were derived from responses to questions relating to dental visiting within the public sector only. Consequently, by not incorporating private dental attendance into the measure of behaviour, and also because references to visiting the dentist privately were excluded from the derivation of the main components of the TPB, the resultant dataset strictly captured patients' perceptions of visiting the dentist within the public dental service and therefore eliminated as much as possible the confounding effects of visiting within the private sector, giving a clearer picture of the influential dental visiting factors specific to the public sector.

### Dental visiting behaviour

This research sought to identify the motivational factors underlying dental visiting behaviour in a sample of users of public dental services. This study was conducted because there was limited evidence of the influence of psychosocial factors and health beliefs on public dental patients' patterns of public dental service use in SA, and more information was needed to help inform dental health policy and the delivery of services. It was found that public dental patients held fairly favourable attitudes toward visiting the dentist, perceived positive social pressure to do so and generally felt in control of visiting the dentist. The association of each of these factors with intention to visit the dentist varied, with correlational analyses showing self-efficacy to be more strongly associated with intention, followed by attitudes toward dental visiting and subjective norms. This research has highlighted the relative importance of the TPB constructs upon behavioural intention and subsequent behaviour. These relationships should be considered when designing educational programs to promote dental attendance. For instance, in order to increase people's motivation/intention to attend dentists, self-efficacy seems to be by far the most important factor to influence, followed in descending order by attitudes and subjective norm. In the behaviour model, both intention and self efficacy had a statistically significant association with dental attendance behaviour, calling for both a motivational and a structural educational approach. Furthermore, because perceived control was not statistically significantly associated with intention, the independent effect of self efficacy upon subsequent behaviour might reflect lack of confidence in ones ability to attend dentists (not perceived controllability or the extent to which attendance is up to the actor) and might call for reduction in structural barriers as a focus for intervention.

### Prediction of intention and behaviour

The use of regression models allowed for the valuation of the importance of each of the constructs of the TPB relative to dental visiting intention and behaviour. The four factors to emerge as important predictors of intention were attitudes, subjective norms, self-efficacy and perceived control. Users of public dental services were less likely to believe that the decision to visit the dentist was under their control (i.e., in terms of perceived difficulties in visiting the dentist), although those intending to visit were more likely to have positive attitudes toward visiting the dentist, perceive support from significant others to visit the dentist and be confident within themselves of their ability to make a visit. These results suggested that although one may hold positive beliefs and attitudes toward visiting the dentist, and despite feeling comfortable in going to the dentist, there are external influences that affect intentions to visit the dentist. As a result, the most effective interventions may be those that attempt to change structural/organisational influences. Direct attempts at encouraging dental visiting will need to involve initiatives that improve access to care, such as addressing the cost of dental care or the long waiting times that currently exist within the public dental system. Whilst this research suggests that these sorts of interventions seem appropriate, there are a number of issues raised by other researchers using this model that should also be considered. These issues relate to the measurement and conceptualisation of the perceived behavioural control construct. Whilst there is agreement about the multidimensionality of this construct (in terms of self-efficacy and perceived control), and indeed the findings from this study support, researchers have questioned whether perceived behavioural control when operationalised in terms of perceived difficulty is just another measure of attitudes, and whether perceived behavioural control when operationalised in terms of self-efficacy can really be discriminated from intentions [39]. Bearing these issues in mind, further work should be carried out to explore the dimensional structure of perceived behavioural control in this context so that appropriate focuses for intervention can be determined.

In this research, a modest amount of the variability in intentions (i.e., 12.0%) could be explained by respective components of the model. The somewhat low predictive power is disappointing since intentions and its predictors were measured at the same time on the same questionnaire using similar items – conditions that should maximise predictive power. The upside however, was that the proximal determinants of intention (as specified by the TPB model) still explained a modest amount of the variation with all of the variables being significant predictors. Modest predictive power for intentions may have been the result of a lack of variation in responses to scales measuring intentions, attitudes, subjective norms and perceived behavioural control and may reflect a bias in the original sample selection. For example, those agreeing to participate in the study may have been those people with fairly, or very strong, positive attitudes. Even the addition of past behaviour as a potential predictor of intentions did not improve the predictive power of the model. Ajzen [17] suggests that when individuals have ambivalent or uncertain attitudes and normative influences, the effect of prior experiences will more strongly affect intentions. In particular, when individuals have no clear plan of action, they are more likely to rely on their experiences to gauge their intentions as well (i.e. residual effects of prior experience can be powerful, particularly in situations where individuals have little certainty in terms of their attitudes, subjective norms, or their perceived behavioural control). As Ajzen [17] suggests about the residual effects of past behaviour on behavioural intent, prior behavioural experience can affect behavioural intent; and yet prior experience may be mitigated by the intervening factors outlined within TPB as seems to be the case in this research. Participants in this study had strong intentions and quite favourable attitudes, subjective norms and perceptions of behavioural control.

The amount of variance explained in actual dental attendance by measures of intention and perceived behavioural control components was small, only 6.6%. The inclusion of past behaviour in the model strengthened the model's predictive power, with the amount of variance explained increasing to 14.1%. During the period of post-questionnaire follow-up only 35.4% of questionnaire respondents visited the dentist, so perhaps those who had yet to visit during the observed follow-up period did in fact intend to visit but simply had not yet done so. In fact, 77.4% reported that they did intend to visit the dentist, but only a small proportion actually did visit. Perhaps a longer follow-up period was needed to better capture the dental behaviour of study participants as longer time intervals allow more opportunities for the behaviour to be performed, increasing the intention-behaviour correspondence [40].

Interestingly, those with a past pattern of emergency dental visiting had significantly greater odds of visiting the dentist post-questionnaire. Perhaps familiarity with visiting the dentist as an emergency dental patient in the past subdued the effect of their perceptions of difficulty associated with visiting the dentist since perceived control failed to be a significant predictor of visiting the dentist. This finding supports Ajzen's [12] assertions about one's perceptions of control being altered once they believe that they are able to perform a particular behaviour.

Further explanations are offered for the modest predictive power for intentions and behaviour. Firstly, a lack of variation in responses to scales directly measuring intention, attitude, subjective norm and perceived behavioural control may have been a contributing factor. This may reflect a bias in the original sample selection. For example, those agreeing to participate in the study may have been those people with fairly, or very strong, positive attitudes and beliefs. Secondly, there was a lack of correspondence between the intention scale, which had 7 points, and the behaviour measure, which had 2 points. It is impossible to have a linear relationship, let alone a perfect linear relationship, and explain all of the variation when there are unequal numbers of scale categories [40]. However, even with equal categories for the two measures, if the distributions are not the same, then it is not possible to obtain a perfect correlation between the two measures [40]. For the dichotomous measures of intention (i.e., intend to visit or do not intend to visit) and behaviour (i.e., visited or did not visit) there was a 23%/77% No/Yes split on intention but a 65%/35% No/Yes split on behaviour. Because the two distributions were not concordant, only a small proportion of the variance in behaviour will be accounted for by intention, explaining why intention, although significant, had low predictive power of actual behaviour in the behaviour model. Thirdly, a high intentions-behaviour association is likely to be obtained if intentions remain stable. However, intentions may change. Sutton [40] explains that the longer the time period between the measurement of intention and behaviour, the greater the probability that unexpected events will occur, leading to changes in intention. This may certainly be the case when measuring one's intention to visit the dentist at a public dental clinic. For instance, an individual may not intend to ever visit the dentist but may develop an unexpected dental problem causing them to make an emergency dental visit. Or perhaps personal circumstances change and they become ineligible to receive public dental care and therefore do not make a dental visit despite their reported intention to do so. Also, Sutton [40] comments that some participants may not be 'engaging in real decision making' when they are completing the questionnaire. If intentions are measured before they have been formed the relationship between intention and behaviour will not be as strong [40].

In the context of the TPB, health behavioural change is the result of reciprocal relationships between the environment, personal factors, and attributes of the behaviour itself. People's perceived control over the opportunities, resources, and skills needed to perform a behaviour affect behavioural intentions and actual performance of the behaviour. In this research, regression analyses highlighted the predictive strength of the self-efficacy and perceived control construct for dental visiting intention and the self-efficacy construct for actual dental visiting behaviour. Based on these analyses, interventions should target individuals' perceptions of behavioural control when seeking to increase dental visiting intentions and promote dental attendance, particularly preventive dental attendance. An approach to enhancing an individual's control over visiting the dentist would be to make changes or intervene at an environmental level. This may involve measures that increase the availability and accessibility of public dental facilities.

The results from this study can also be used in patient- and community-centred health education by identifying and enhancing the psychological features (such as self-efficacy) that characterise dental visiting behaviours. Perceptions of self-efficacy can be used to explain behavioural changes, to predict effects of interventions, and to improve dental health behaviour. In relation to dental health behaviour, self-efficacy determines whether a given behaviour is initiated (as demonstrated by the behaviour models) and for how long the behaviour may continue against any obstacles that are encountered. This is because self-efficacy beliefs provide 'the foundation for human motivation, well-being, and personal accomplishment' [41]. There is little incentive for individuals to carry out a behaviour, or to persevere in the face of difficulties and barriers, if they believe that the behaviour will not lead to outcomes they desire [41]. In addition, the results suggest that attitudinal considerations and beliefs regarding other people's support of the behaviour also have a role to play in dental visiting intention.

Effective interventions for behavioural change must therefore influence multiple levels because, as this research has demonstrated, dental health behaviour is shaped by many environmental subsystems, including family, community, beliefs, organisation of dental services, and the physical and social environments in which people live.

## Conclusion

Public dental service utilisation appeared to be hindered by perceived barriers to dental care. Efforts should therefore be directed at reducing barriers that currently exist. This may improve access to public dental services for many eligible adults. This research can assist in identifying the necessary targets for intervention to positively affect the dental visiting behaviour of disadvantaged groups of adults within the Australian population, and can be used to promote more effective and efficient oral health care to those in need.

## Competing interests

The authors declare that they have no competing interests.

## Authors' contributions

LL and AJS conceived of the research problem. AJS supervised the research. LL planned and conducted the analysis. LL drafted the manuscript, and AJS participated in completing the manuscript. All authors read and approved the manuscript.

## Pre-publication history

The pre-publication history for this paper can be accessed here:


